# Recent Update on Radiation Dose Assessment for the State-of-the-Art Coronary Computed Tomography Angiography Protocols

**DOI:** 10.1371/journal.pone.0161543

**Published:** 2016-08-23

**Authors:** Sock Keow Tan, Chai Hong Yeong, Kwan Hoong Ng, Yang Faridah Abdul Aziz, Zhonghua Sun

**Affiliations:** 1 Department of Biomedical Imaging, Faculty of Medicine, University of Malaya, 50603 Kuala Lumpur, Malaysia; 2 Department of Medical Radiation Sciences, Curtin University, Perth, WA 6845, Australia; Maastricht University Medical Centre, NETHERLANDS

## Abstract

**Objectives:**

This study aimed to measure the absorbed doses in selected organs for prospectively ECG-triggered coronary computed tomography angiography (CCTA) using five different generations CT scanners in a female adult anthropomorphic phantom and to estimate the effective dose (H_E_).

**Materials and Methods:**

Prospectively ECG-triggered CCTA was performed using five commercially available CT scanners: 64-detector-row single source CT (SSCT), 2 × 32-detector-row-dual source CT (DSCT), 2 × 64-detector-row DSCT and 320-detector-row SSCT scanners. Absorbed doses were measured in 34 organs using pre-calibrated optically stimulated luminescence dosimeters (OSLDs) placed inside a standard female adult anthropomorphic phantom. H_E_ was calculated from the measured organ doses and compared to the H_E_ derived from the air kerma-length product (*P*_KL_) using the conversion coefficient of 0.014 mSv∙mGy^-1^∙cm^-1^ for the chest region.

**Results:**

Both breasts and lungs received the highest radiation dose during CCTA examination. The highest H_E_ was received from 2 × 32-detector-row DSCT scanner (6.06 ± 0.72 mSv), followed by 64-detector-row SSCT (5.60 ± 0.68 and 5.02 ± 0.73 mSv), 2 × 64-detector-row DSCT (1.88 ± 0.25 mSv) and 320-detector-row SSCT (1.34 ± 0.48 mSv) scanners. H_E_ calculated from the measured organ doses were about 38 to 53% higher than the H_E_ derived from the *P*_KL_-to-H_E_ conversion factor.

**Conclusion:**

The radiation doses received from a prospectively ECG-triggered CCTA are relatively small and are depending on the scanner technology and imaging protocols. H_E_ as low as 1.34 and 1.88 mSv can be achieved in prospectively ECG-triggered CCTA using 320-detector-row SSCT and 2 × 64-detector-row DSCT scanners.

## Introduction

According to the latest update published by the American Heart Association [1], cardiovascular disease (CVD) is the leading global cause of death, accounting for 17.3 million deaths per year. It is the first killer of the population in the United States, taking more lives than all forms of cancer combined. While invasive coronary angiography remains as the gold standard for the diagnosis of coronary artery diseases (CAD), its associated costs and morbidity including a 1.7% rate of major complications have led to the development of non-invasive imaging modalities [2]. Coronary computed tomography angiography (CCTA) is a well-established imaging technique that has high per-patient sensitivity (99%), positive predictive value (92%) and negative predictive value (95%) for obstructive CAD [3].

CCTA was first approved by the U.S. Food and Drug Administration (FDA) in 2004 using 64-slice CT. The 64-slice per gantry rotation can be achieved using either 64-detector-row, or 32-detector-row with a strategy to double the slice number by alternating the focal spot of the X-ray source [4]. The technology has then rapidly evolved from 64-slice to 128-, 256-, 320- and the recent 640-slice CT to achieve better spatial resolution, temporal resolution, larger volume coverage and lower radiation dose to the patients. As motion artifact (due to rapid heart beat) is one of the most significant challenges in CCTA, temporal resolution of less than 100 ms is usually desirable. Temporal resolution of a single X-ray tube corresponds to approximately half of the gantry rotation time (typically 330 ms). Further improvement of temporal resolution has been achieved in 128- and 256-detector-row CT scanners, with gantry rotation time ranged between 270 and 280 ms. With the introduction of dual-source CT (DSCT), temporal resolution can be further improved from 165 to 83 ms. High diagnostic accuracy (93%), sensitivity (94%) and negative predictive value (97%) have been reported in CCTA using 2 × 64-detector-row DSCT scanner [5]. Being another latest scanner version for CCTA, the 320-detector-row SSCT provides the largest z-coverage per gantry rotation (160 mm), sufficiently covering the whole heart at one rotation. This configuration allows 3-dimensional volumetric heart imaging to be carried out within diastole of one R-R interval [6]. In addition, 4-dimensional CT or volumetric cine imaging is possible if the X-ray beam is turned on for a longer period to capture the heart over one or more cardiac cycles [7]. Other proposed methods to overcome motion-induced image degradation include an opening of the padding (adding surrounding X-ray beam time to the mid-diastolic window with retrospective gating), multi-segmental reconstruction and motion correction algorithm [8–10]. Padding with retrospective gating and multi-segmental reconstruction are associated with substantial increase of patient dose. Fuchs et al. [8] have reported image quality improvement and interpretability of prospectively ECG-triggered CCTA with motion correction algorithm at average heart rate of 69 ± 9 beats per minute (bpm).

While conventional angiography may expose the patient with radiation dose in the range of 3 to 9 mSv, effective dose (H_E_) as high as 12 to 21 mSv have been reported in CCTA using 64-detector-row CT scanners [11, 12]. With the later generation CT scanners (higher than 64-detector-row), H_E_ as low as 0.4 to 1.2 mSv can be achieved for an average sized patient in prospectively ECG-triggered CCTA [13–16]. Although several clinical studies have been conducted to assess radiation dose during prospective ECG-triggered CCTA, the data mainly rely on the air kerma-length product (*P*_KL_) reported in the CT console [17, 18]. It is indeed important to assess the radiation dose imparted to the specific organs that are being exposed, such as breasts, lungs, heart, liver, stomach, etc. However, to the best of our knowledge, research in this area is scare, and this is the main reason for us to conduct this study to fill this gap in the current literature.

This study therefore aimed to assess the radiation dose received from prospectively ECG-triggered CCTA using different generations of CT scanners through direct measurement of organ doses in a standard female adult anthropomorphic phantom. We hypothesized that there exists wide variation between radiation dose associated with CCTA acquired with different generation of scanners.

## Materials and Methods

### Study Design

This study was designed to measure organ doses received from a prospectively ECG-triggered CCTA examination using a standard female adult anthropomorphic phantom and optically stimulated luminescence dosimeters (OSLDs). Dose measurement was carried out using five CT scanners of different generations located at five different centers. The recommended CCTA imaging protocols were used according to the manufacturers’ guidelines.

#### Anthropomorphic Phantom and OSLDs

**Fig 1:**
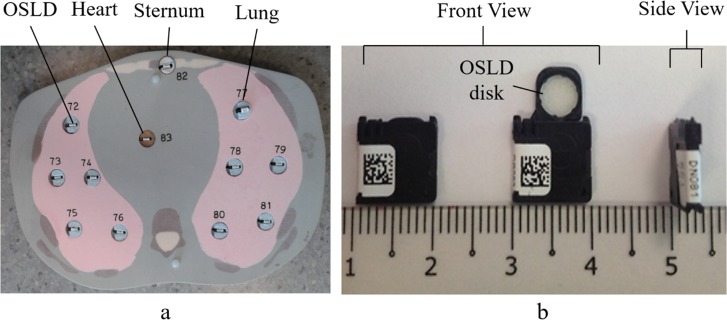
(a) Axial view of the phantom’s sectional slab showing the lungs, spine, heart and sternum. The OSLDs are loaded into the tissue-equivalent plugs within the organs. (b) Front and side views of OSLD’s holder.

A female adult anthropomorphic phantom (702-G, CIRS Inc., Norfolk, Virginia, USA) assembled with multiple holes for the placement of the OSLDs (NanoDot, Landauer Inc., Glenwood, IL) was used. The phantom represented a female adult of 160 cm height and 55 kg weight. The phantom is made of tissue-equivalent materials that simulate average soft tissues, average bone tissues, cartilage, spinal cord and disks, lung, brain and sinus, where the linear attenuation coefficient of the materials are within 3% of the actual tissues for photon energies ranged 40 to 150 keV [19]. The phantom is sectioned into 38 contiguous slabs of 25 mm thickness. Each section contains several 14 mm-diameter holes and plugs for OSLDs placement across 19 organs (Fig 1A). The phantom has a pair of detachable breasts with base diameter 10.8 cm and height 4.3 cm. The ratio of glandular: adipose tissues is 50: 50. Specific holes and plugs are located in the breasts at 1 cm below the skin surface for OSLD placement.

The OSLD is made of aluminum oxide doped with carbon (*Al*_2_*O*_3_:*C*). It is in disk-shaped of 5 mm diameter and 0.2 mm thickness, wrapped in a light-tight 10 × 10 × 2 mm^3^ black plastic carrier with a density of 1.03 g.cm^-^³ (Fig 1B). The OSLDs used in this study were calibrated for X-ray energy of 120 kVp. A calibrated OSLD reader system (MicroStar InLight reader, Landauer, Glenwood, Illinois, USA) was used to acquire the energy released by each OSLD and subsequently converted it to absorbed dose (mGy) based on the calibration curve.

#### CT Scanners and Imaging Protocols

The five different generations CT scanners used in this study include 64-detector-row single source CT (SSCT) system (Optima CT 660, GE Healthcare, USA), 64-detector-row SSCT system (Ingenuity 128, Philips Healthcare, USA), 2 × 32-detector-row dual source CT (DSCT) system (Somatom Definition Dual Source, Siemens Healthcare, Germany), 2 × 64-detector-row DSCT system (Somatom Definition Flash, Siemens Healthcare, Germany) and 320-detector-row SSCT system (Aquilion ONE, Toshiba Medical System, Japan). The prospectively ECG-triggered CCTA imaging protocols recommended by the respective CT manufacturers were used. The protocols include Snapshot Pulse Acquisition (Optima CT 660, GE Healthcare, USA)–thereafter referred as “protocol A”, Step and Shoot Cardiac Acquisition (Ingenuity 128, Philips Healthcare, USA)–thereafter referred as “protocol B”, Adaptive Cardio Sequence Acquisition (Somatom Definition Dual Source, Siemens Healthcare, Germany)–thereafter referred as “protocol C”, Flash Spiral Acquisition (Somatom Definition Flash, Siemens Healthcare, Germany)–thereafter referred as “protocol D”, and Volumetric Cardiac Acquisition (Aquilion ONE, Toshiba Medical Centre, Japan)–therefore referred as “protocol E”.

**Fig 2:**
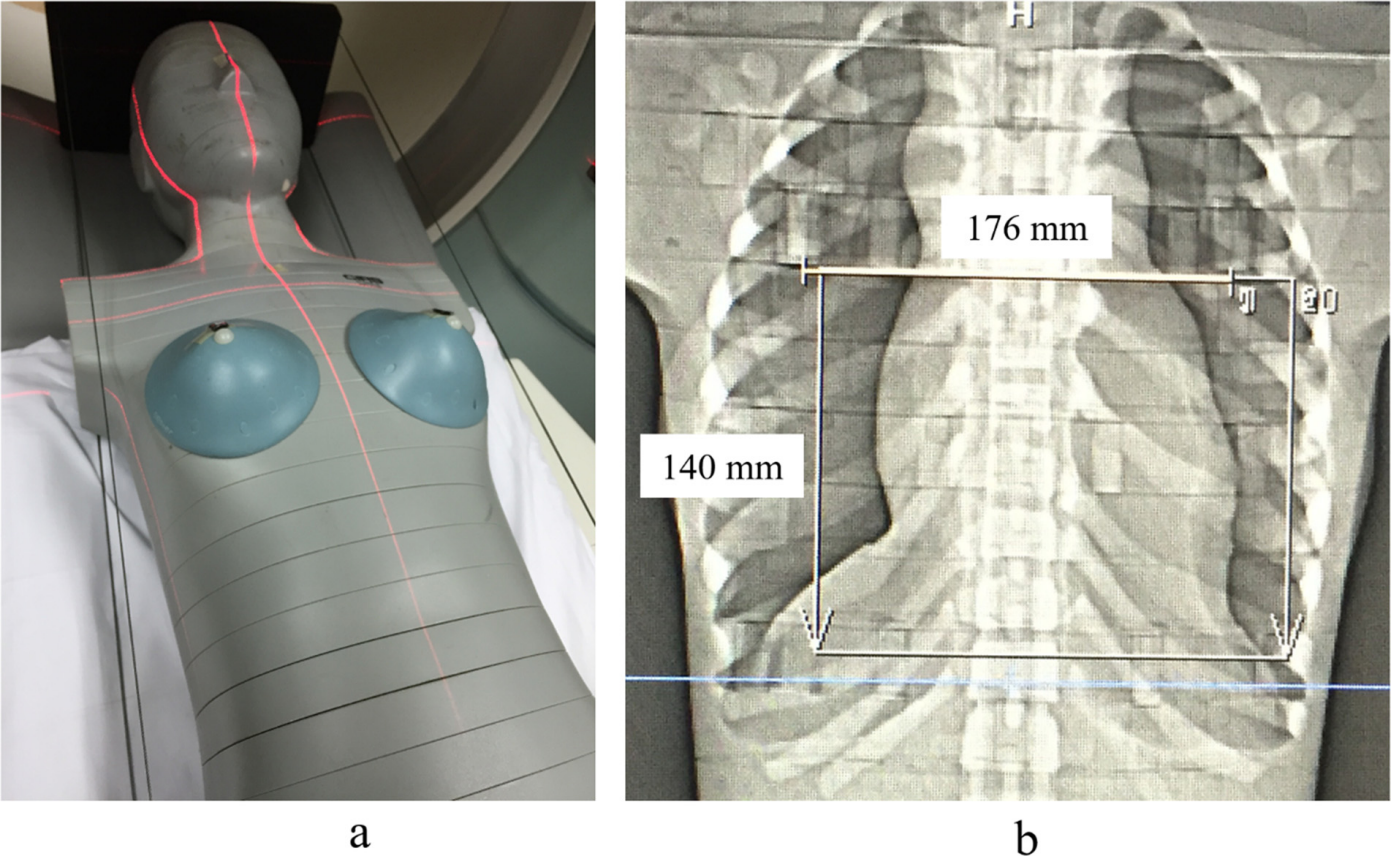
a) Positioning of phantom according to the clinical CCTA settings; b) SPR image of phantom with the scan range planned for CCTA (white box).

The anthropomorphic phantom pre-loaded with 244 OSLDs from brain to femora was positioned on the CT scanner table (Fig 2A). The scan range was fixed at 140 mm covering from the carina of trachea to the apex of the heart (Fig 2B). The CT scanner was connected to an ECG monitor and a constant heart rate of 60 bpm was applied using ECG demo mode. Table 1 summarizes the scanning parameters for a complete CCTA examination including the scan projection radiograph (SPR), bolus tracking or test bolus and prospectively ECG-triggered CCTA using the respected CT scanners. For bolus tracking technique, threshold of 150 HU was set at the region of interest (ROI) to initiate the scan. For the test bolus technique, six exposures were performed at the ROI to identify the triggering threshold and continued with the prospectively ECG-triggered CCTA.

**Table 1:** Scanning parameters for prospectively ECG-triggered CCTA using five CT scanners from different generations.

Imaging Protocol	Protocol A	Protocol B	Protocol C	Protocol D	Protocol E
**Scanner model**	Optima CT 660	Ingenuity128	SomatomDefinition Dual Source	Somatom Definition Flash	Aquilion ONE
**Number of slices**	128	128	128	256	640
**Detector type**	HiLight V-Res VolaraDAS	NanoPanel	Ultrafast ceramic	Ultrafast ceramic	Solid-stateGd_2_O_2_S
**Detector-row**	64	64	2 × 32	2 × 64	320
**Detector thickness (mm)**	0.625	0.625	0.6	0.6	0.5
**Z-coverage per gantry rotation (mm)**	40.0	40.0	19.2	38.4	160.0
**Gantry rotation time (ms)**	350	300	330	280	350
**Scan Projection Radiograph (SPR)**					
**Tube voltage (kVp)**	120	120	120	120	120
**Tube current (mA)**	40	30	35	50	50
**Bolus tracking/Test bolus**					
**Tube voltage (kVp)**	120	120	120	120	120
**Tube current-time (mAs)**	40	30	45	60	25
**Contrast timing method**	Bolus tracking	Bolus tracking	Test bolus	Test bolus	Test bolus
**Number of scan**	6	6	6	6	6
**Threshold (HU)**	150	150	-	-	-
**Scanning time (s)**	8.76	10.0	10.5	10.3	10.0
**Prospectively ECG-triggered CCTA**					
**Acquisition technique**	Snapshot Pulse	Step and Shoot Cardiac	Adaptive Cardio Sequence	Flash Spiral	Volumetric Cardiac
**Tube voltage (kVp)**	120	120	120	120	120
**Tube current-time (mAs)**	197	180	218	169	15
**Heart rate (bpm)**	60	60	60	60	60
**Tube rotation time (s)**	0.35	0.40	0.38	0.28	0.35
**Total exposure time (s)**	1.76	1.96	3.04	0.45	1.22
**Acquisition slice thickness (mm)**	0.625	0.625	0.6	0.6	0.5
**Reconstruction slice thickness (mm)**	0.625	0.9	3.0	0.75	0.5

#### Organ Dose Measurement

A total of three measurements were done for each imaging protocol. Each measurement was obtained by averaging the results from five exposures. The OSLD signals were analyzed and converted to absorbed dose using the calibration curve. Organ doses were obtained by multiplying the absorbed dose with individual tissue weighting factors recommended by the International Commission on Radiological Protection Publication (ICRP) Publication 103 [20].

#### Effective Dose (HE) Estimation

The H_E_ was estimated using two different approaches in this study and the results were compared. First, the H_E_ was computed by summing up all the organ doses measured from the anthropomorphic phantom. Second, the H_E_ was calculated by multiplying the air kerma-length product (*P*_KL_) (previously known as dose length product) recorded from the CT console with the E_KL_ conversion factor as following [20, 21]:

$$H_{\text{E}}={\text{E}}_{\text{KL}}P_{KL}$$

Where E_KL_ is region-specific, *P*_KL_ normalized H_E_ (mSv.mGy^-1^cm^-1^) conversion factor. The E_KL_ for chest, 0.014 mSv.mGy^-1^cm^-1^ as recommended by the European Commission (EC) and Public Health England (PHE) (formerly National Radiological Protection Board (NRPB)) was used in this study [22, 23].

#### Statistical Analysis

The statistical analysis was performed using a commercially available software package (IBM SPSS Statistical 20.0, SPSS Inc, Chicago, USA). Continuous variables were presented as mean ± standard deviation. The organ doses measured from all the protocols were compared using one-way ANOVA, followed by post-hoc Fisher’s LSD test to identify the significance of the differences between each data pair. 95% confidence interval was used in all the statistical tests.

## Results

### Organ Doses

**Fig 3:**
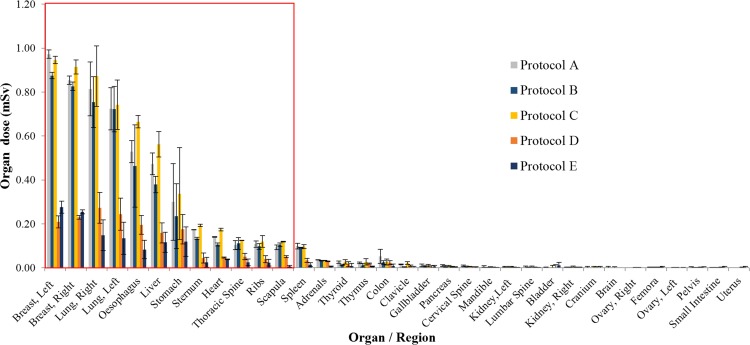
Graph shows the organ dose of 34 organs obtained using prospectively ECG-triggered CCTA in five different generations CT scanners. The red box indicates organs included in the scanning field of view.

**Fig 4:**
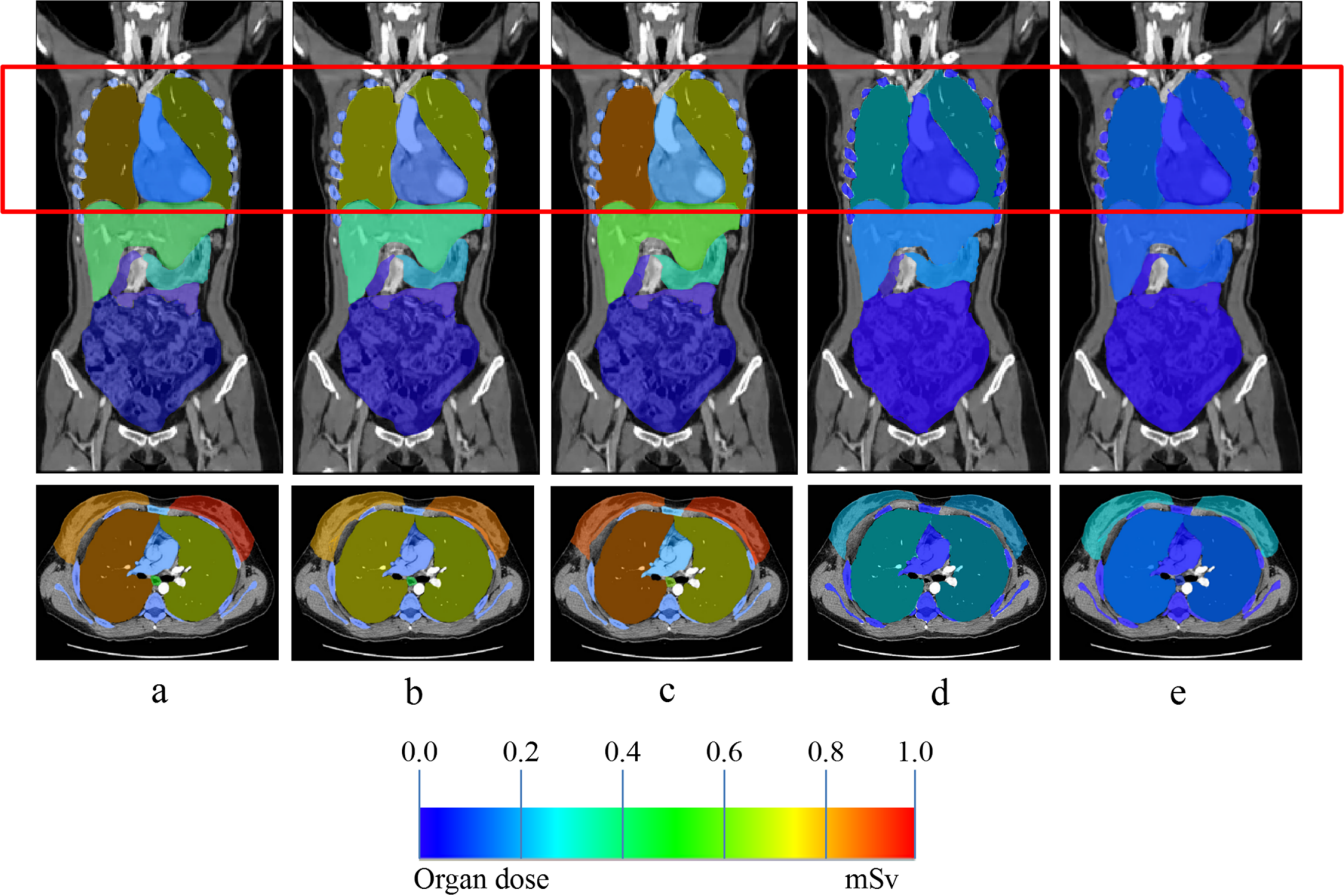
Organ dose obtained in prospectively ECG-triggered CCTA using a) Protocol A; b) Protocol B; c) Protocol C; d) Protocol D; e) Protocol E (from left to right).

The organ doses measured from the anthropomorphic phantom are tabulated in Table 2. There were 34 organs involved from brain to femora excluding skin. Comparison of organ doses across different scanners is better presented in a graph format, as shown in Fig 3. Ten organs were directly exposed to the primary beam in the field of view (FOV) during CCTA, i.e. breasts, lungs, oesophagus, liver, stomach, sternum, heart, thoracic spine, ribs and scapula. Among these organs, breasts received the highest radiation dose followed by lungs, oesophagus, liver, stomach, etc. Using 320-detector-row SSCT scanner and Protocol E, the organ doses were significantly reduced compared to all other scanners and protocols. The second lowest radiation dose was achieved by using 2 × 64-detector-row DSCT scanner and protocol D, followed by 64-detector-row SSCT scanner with Protocol B and Protocol A. The 2 × 32-detector-row DSCT scanner contributed higher dose compared to the 64-detector-row SSCT. One-way ANOVA test shows significant difference (p < 0.05) for organ doses measured in different protocols. On post-hoc Fisher’s LSD test, organ doses measured in protocol E was statistically different to organ doses measured in protocols A, B and C; organ doses measured in protocol D was statistically significant different to organ doses measured in protocol A and C, while no other comparison was statistically significant different (Table 3). Fig 4 illustrates the distribution of dose at different organs at a glance. The colour coding indicates the level of radiation dose received by the respective organs, and the red box shows the FOV. Protocol E contributed the least dose to all organs among all the protocols.

**Table 2:** Mean organ doses measured from the female anthropomorphic phantom during prospectively ECG-triggered CCTA.

Organ	Mean Absorbed Dose (mGy)				
	Protocol A	Protocol B	Protocol C	Protocol D	Protocol E
**Adrenals **	3.96 ± 0.05	3.46 ± 0.16	3.72 ± 0.04	3.20 ± 0.02	0.83 ± 0.13
**Bladder**	0.05 ± 0.06	0.01 ± 0.02	0.11 ± 0.17	0.22 ± 0.01	0.30 ± 0.33
**Brain**	0.36 ± 0.30	0.01 ± 0.02	0.26 ± 0.20	0.04 ± 0.02	0.04 ± 0.07
**Breast, Left**	16.20 ± 0.32	14.58 ± 0.24	15.76 ± 0.28	3.50 ± 0.02	4.60 ± 0.45
**Breast, Right**	14.23 ± 0.32	13.77 ± 0.31	15.23 ± 0.53	3.83 ± 0.06	4.24 ± 0.14
**Cervical Spine**	0.77 ± 0.35	0.29 ± 0.41	0.62 ± 0.01	0.29 ± 0.20	0.29 ± 0.19
**Clavicle**	1.43 ± 0.14	0.37 ± 0.13	1.95 ± 0.54	0.86 ± 0.06	0.25 ± 0.32
**Colon**	0.43 ± 0.27	0.20 ± 0.07	0.22 ± 0.11	0.20 ± 0.01	0.06 ± 0.08
**Cranium**	0.29 ± 0.30	0.01 ± 0.01	0.27 ± 0.30	0.03 ± 0.01	0.20 ± 0.38
**Femora**	0.12 ± 0.17	0.01 ± 0.02	0.17 ± 0.19	0.01 ± 0.00	0.27 ± 0.39
**Gallbladder**	1.16 ± 0.69	0.82 ± 0.30	1.11 ± 0.54	0.55 ± 0.32	0.64 ± 0.56
**Heart**	15.28 ± 0.18	11.51 ± 0.78	18.91 ± 0.66	5.14 ± 0.41	4.17 ± 0.23
**Kidney, Left**	0.95 ± 0.51	0.87 ± 0.51	0.95 ± 0.35	0.41 ± 0.11	0.29 ± 0.20
**Kidney, Right**	0.60 ± 0.29	0.62 ± 0.25	0.90 ± 0.80	0.57 ± 0.27	0.37 ± 0.44
**Liver**	11.83 ± 1.22	9.47 ± 0.92	14.06 ± 1.45	3.98 ± 0.17	2.91 ± 1.15
**Lumbar Spine**	0.31 ± 0.38	0.15 ± 0.14	0.40 ± 0.30	0.20 ± 0.27	0.07 ± 0.06
**Lung, Left**	12.06 ± 1.61	12.04 ± 1.72	12.36 ± 1.88	4.07 ± 0.47	2.25 ± 1.22
**Lung, Right**	13.57 ± 2.04	12.57 ± 1.94	14.54 ± 2.30	4.54 ± 0.54	2.48 ± 1.17
**Mandible**	0.57 ± 0.38	0.06 ± 0.11	0.43 ± 0.09	0.19 ± 0.03	0.05 ± 0.09
**Oesophagus**	13.21 ± 1.26	11.58 ± 4.69	16.63 ± 0.69	6.89 ± 3.25	2.08 ± 1.05
**Ovary, Left**	0.02 ± 0.01	0.05 ± 0.00	0.04 ± 0.01	0.02 ± 0.00	0.03 ± 0.01
**Ovary, Right**	0.01 ± 0.01	0.01 ± 0.01	0.05 ± 0/01	0.02 ± 0.00	0.01 ± 0.02
**Pancreas**	1.15 ± 0.37	0.66 ± 0.47	0.96 ± 0.31	0.55 ± 0.06	0.41 ± 0.21
**Pelvis**	0.22 ± 0.21	0.07 ± 0.08	0.11 ± 0.10	0.05 ± 0.02	0.14 ± 0.31
**Ribs**	9.90 ± 1.37	8.92 ± 1.17	11.00 ± 2.47	3.62 ± 0.42	2.13 ± 1.43
**Scapula**	8.60 ± 1.00	9.62 ± 0.79	10.94 ± 0.12	4.64 ± 0.65	0.62 ± 0.40
**Small Intestine**	0.07 ± 0.08	0.07 ± 0.10	0.07 ± 0.07	0.05 ± 0.03	0.27 ± 0.40
**Spleen**	10.74 ± 1.44	9.99 ± 0.12	10.44 ± 1.03	3.70 ± 0.14	1.58 ± 0.93
**Sternum**	15.88 ± 0.09	12.23 ± 0.52	17.69 ± 0.47	4.11 ± 0.11	2.28 ± 2.06
**Stomach**	2.50 ± 1.45	1.96 ± 1.23	2.81 ± 1.74	1.46 ± 0.91	0.99 ± 0.56
**Thoracic Spine**	9.52 ± 1.88	10.21 ± 2.42	11.45 ± 0.07	4.71 ± 1.68	2.33 ± 1.38
**Thymus**	2.47 ± 0.47	1.26 ± 0.89	2.93 ± 1.55	2.10 ± 0.77	0.77 ± 0.18
**Thyroid**	0.65 ± 0.14	0.31 ± 0.06	0.68 ± 0.26	0.42 ± 0.04	0.21 ± 0.29
**Uterus**	0.01 ± 0.01	0.01 ± 0.02	0.04 ± 0.04	0.03 ± 0.01	0.29 ± 0.38

**Table 3:** Results of post-hoc Fisher’s LSD test to evaluate significance level of each protocol pair.

Data-Pair	P-value
**Protocol A–Protocol B**	0.750
**Protocol A–Protocol C**	0.799
**Protocol A–Protocol D**	0.047*
**Protocol A–Protocol E**	0.023*
**Protocol B–Protocol C**	0.567
**Protocol B–Protocol D**	0.094
**Protocol B–Protocol E**	0.050
**Protocol C–Protocol D**	0.025*
**Protocol C–Protocol E**	0.012*
**Protocol D–Protocol E**	0.774

* P < 0.05 is considered statistically significant different.

### H_E_ Estimation

The comparison of H_E_ obtained by summing up all the organ doses from the phantom measurement (measured H_E_) and by computing using the *P*_KL_-to-H_E_ conversion factor (computed H_E_) is shown in Table 4. In general, the measured H_E_ was higher than the computed H_E_ by 38.3 to 53.2%. Protocol C contributed the highest H_E_, followed by protocol A, B, D and E.

**Table 4:** Estimated effective doses obtained from prospectively ECG-triggered CCTA using different generations CT scanners and protocols.

Parameter	Protocol A	Protocol B	Protocol C	Protocol D	Protocol E
***P***_**KL**_ **(mGy.cm)**	193.40 ± 2.52	168.10 ± 3.44	204.00 ± 3.30	83.00 ± 3.01	57.90 ± 1.21
**Measured H**_**E**_ **(mSv)**	5.60 ± 0.68	5.02 ± 0.73	6.06 ± 0.72	1.88 ± 0.25	1.34 ± 0.48
**Computed H**_**E**_ **(mSv)**	2.71 ± 0.04	2.35 ± 0.05	2.86 ± 0.05	1.16± 0.04	0.81 ± 0.02
**% difference (Measured H**_**E**_**−Computed H**_**E**_**)**	51.6%	53.2%	52.8%	38.3%	39.6%

## Discussion

To our knowledge, this is the first report comparing the direct measured organ doses from prospectively ECG-triggered CCTA using 64-, dual source 2 × 32-, dual source 2 × 64- and 320-detector-row CT scanners and a standard female adult anthropomorphic phantom. The dose measurement setup in this study followed exactly the procedures of a typical CCTA examination of a female patient, that include the positioning, scout scanning (or scan projection radiograph), bolus tracking or test bolus and prospectively ECG-triggered CCTA imaging. The imaging parameters recommended by different CT scanner manufacturers are used according to the CT scanner model. Low heart rate of 60 bpm was used during ECG-triggered CCTA considering that this is the average heart rate in most of the clinical cases after beta blocker is applied. Low heart rate is desirable to guarantee a better image quality and lower radiation dose to the patient during prospectively ECG-triggered CCTA [7].

The data from this study show that, if excluding skin, breasts received the highest radiation dose, followed by lungs, oesophagus, liver, stomach, sternum and heart. It is therefore important to note that, although heart is the organ of interest in CCTA imaging, other organs such as breasts, lungs, oesophagus, liver and stomach receive relatively higher radiation dose due to their higher sensitivity towards ionizing radiation. According to ICRP-103 publication, heart is one of the most radioresistant organs which are categorized as “remainder tissues” when considering its tissue weighting factor. Although spleen was not included in the FOV, it still received comparable dose as the scapula, ribs and thoracic spine due to scattered radiation from the nearest organ such as liver. The scattered radiation doses received by other organs were negligible.

Among all the CT scanners, 320-detector-row SSCT system gave lowest radiation dose to most of the organs. The measured H_E_ was 1.34 ± 0.48 mSv while computed H_E_ was 0.81 ± 0.02 mSv. It is the current latest CT system that has wide z-axis coverage of 160 mm, enabling the whole heart to be imaged in a single tube rotation. This configuration allows volumetric whole heart imaging during the diastole of one R-R interval and the entire heart is imaged without temporal delay [7]. However, the scanner has a standard temporal resolution of approximately 175 ms which is inferior to the 83 ms from DSCT, therefore, this type of scanner is only suitable to image patients with low and regular heart rate. The recently developed Revolution CT by GE medical system shows promise in imaging patients with high heart rate as it has 160 mm detector array and improved temporal resolution of 140 ms [24].

Although the two models of 64-detector-row SSCT scanners and one model of 2 × 32-detector-row DSCT scanner used in this study have the same total number of detector row, the radiation doses contributed by the SSCT scanners were generally lower than the DSCT scanner by 7 and 17%, respectively. This may be due to the wider z-coverage per gantry rotation (40 mm) in 64-detector-row SSCT scanners, compared to only 19.2 mm z-coverage per gantry rotation in 2 × 32-detector-row DSCT scanner. Consequently, the 2 × 32-detector-row DSCT scanner requires more than 2 times acquisition time in order to achieve the same volume coverage. Since there is slight overlap in each acquisition slice (helical scan), this may result in higher dose. Fortunately, this first generation DSCT scanner has now been replaced by the second generation DSCT scanner. Several improvements have been introduced in the second generation DSCT system. First, the detector row was increased from 2 × 32-detector-row to 2 × 64-detector-row with z-coverage of 38.4 mm. Second, the gantry rotation speed was boosted to 280 ms compared to 330 ms in the first generation system. Third, the scan field-of-view (in the x/y plane) of the second detector (Detector B) was widened from 26 to 33 cm to provide better coverage of patient anatomy. Fourth, a new tin-based selective photon shield was used to filter unnecessary low energy photons from the high energy X-ray tube spectrum. This helps to reduce patient dose and enables the separation of high energy and low energy X-ray spectra during dual-energy imaging. Finally, a new “Flash” scanning mode was introduced in the system, which uses fast gantry rotation time in conjunction with a high table pitch of up to 3.2. With such high pitch, the system can acquire cardiac images in a quarter of heartbeat or 250 ms in a single diastolic phase, compared to scanners that may require several cardiac cycles for image acquisition, hence eliminating additional radiation dose from overlapping slices. In this study, it was observed that, although the H_E_ obtained from the 2 × 64-detector-row DSCT scanner was higher than the 320-detector-row SSCT scanner, the doses delivered to the breasts were actually lower. This was a promising result as breast is one of the most radiosensitive organs in CCTA examination.

In the comparison of measured versus computed H_E_, the mean difference observed from this study ranged between 38.3 and 53.2%. These findings were consistent with the findings from Hurwitz et al where the measured H_E_ were higher than the computed H_E_ [25]. In this study, the latest *P*_KL_-to-H_E_ conversion factor as recommended by the EC and PHE was applied [22, 23]. In our opinion, the measured H_E_ were more reliable than computed H_E_ because the radiation doses were directly measured from all the organs, including those located outside of the primary beam during the CCTA imaging. The use of *P*_KL_-to-H_E_ conversion factor of 0.014 mSv.mGy^-1^cm^-1^ may underestimate the overall radiation exposure from CCTA imaging, hence this method may need to be reviewed and improved. In fact, Gosling et al. [26] and Akmal et al. [18] have both suggested that a conversion factor of 0.028 mSv.mGy^-1^cm^-1^ would give a better estimation of the H_E_ in cardiac-specific imaging.

From our results, the 2 × 32-detector-row DSCT scanner contributed highest H_E_ in prospectively ECG-triggered CCTA, followed by 64-detector-row SSCT scanners, 2 × 64-detector-row DSCT scanner and 320-detector-row SSCT scanner. Although the H_E_ varied from 1.34 ± 0.48 to 6.06 ± 0.72 mSv among different generations of CT scanners and imaging protocols, the radiation doses were relatively low compared to many other CT examinations. A study carried out by Akmal et al. [18] found no significant difference in the H_E_ between genders, however body mass index (BMI) is identified as the main factor that significantly affects the radiation dose. This is also confirmed by a recent study using latest CT model [24].

The radiation doses reported in this study provide medical practitioners with data that can be used to assess risk versus benefit of CCTA examination in patients. However, our study has some limitations. First, since this was a phantom study, only one body type was used. The actual doses will vary from patient to patient, depending on patient body habitus, tube current setting, heart rate and z-axis coverage. Second, only five most commonly used CT scanner models for CCTA examination were used in this study, while the most recent CT scanners such as 128-detector-row SSCT scanner, 256-detector-row SSCT scanner, third generation of DSCT scanner and second generation of 320-detetor-row SSCT scanner were not included in our data acquisition because the latest CT scanners are not available yet in many clinical centers [27].

Hou et al. [16] reported H_E_ of 1.21 ± 0.41 mSv in prospectively ECG-triggered CCTA using 128-detector-row CT scanner. For 256-detector-row CT scanner, H_E_ ranged from 0.18 to 1.22 mSv was reported in prospectively ECG-triggered CCTA at a cut-off heart rate of 67 bpm [28]. Gordic et al. [29] reported that third generation DSCT scanner in high-pitch mode allows diagnostic image quality and H_E_ of 0.4 mSv at heart rate up to 70 bpm in prospectively ECG-triggered CCTA. The authors further concluded that heart rate viability is not significantly related to image quality of CCTA. Using second generation DSCT scanner, Scharf et al. [30] reported that an average heart rate less than 64 bpm is required to obtain the diagnostic depiction of coronary arteries for patients. The better image quality at lower radiation dose in patients with elevated heart rate (70 bpm versus 64 bpm) in third generation DSCT scanner is due to the increase of detector row (2 × 96) with z-coverage of 57.6 mm, gantry rotation speed of 250 ms, scan field-of-view of 50 cm and high pitch scanning. For second generation 320-detector-row SSCT scanner, estimated H_E_ of 2.1 and 2.8 mSv were reported for patient with heart rate < 65 and ≥ 65 bpm, respectively [31]. Finally, we did not include assessment of image quality in this study as our focus is to compare the radiation dose among these different CT scanners. Recent developments in CCTA (both prospectively and retrospectively ECG-triggering) with use of iterative reconstruction (IR) algorithms have been shown to significantly improve image quality while reducing radiation dose to a greater extent [32, 33]. Thus, further studies with testing of these IR techniques on different CT scanners are needed.

## Conclusion

This study provides the most recent data on specific organ dose measurement and H_E_ estimation from prospectively ECG-triggered CCTA examination using five commonly used different generations CT scanners and imaging protocols. Although the heart is the organ of interest in CCTA imaging, breasts and lungs received the highest radiation dose due to their high radiosensitivity towards ionizing radiation. The use of CCTA especially in young women should be considered carefully in conjunction with clinical indications, benefits versus risks and alternative imaging modalities.
